# Controllable growth of ZnO nanorod arrays with different densities and their photoelectric properties

**DOI:** 10.1186/1556-276X-7-246

**Published:** 2012-05-06

**Authors:** Shujie Wang, Chongshun Song, Ke Cheng, Shuxi Dai, Yayan Zhang, Zuliang Du

**Affiliations:** 1 Key Laboratory for Special Functional Materials, Henan University, Kaifeng 475004, People’s Republic of China

**Keywords:** ZnO nanorod arrays, EBL, Hydrothermal, Photoelectric

## Abstract

Since the photoelectric response and charge carriers transport can be influenced greatly by the density and spacing of the ZnO nanorod arrays, controlling of these geometric parameters precisely is highly desirable but rather challenging in practice. Here, we fabricated patterned ZnO nanorod arrays with different densities and spacing distances on silicon (Si) substrate by electron beam lithography (EBL) method combined with the subsequent hydrothermal reaction process. By using the EBL method, patterned ZnO seed layers with different areas and spacing distances were obtained firstly. ZnO nanorod arrays with different densities and various morphologies were obtained by the subsequent hydrothermal growth process. The combination of EBL and hydrothermal growth process was very attractive and could make us control the geometric parameters of ZnO nanorod arrays expediently. Finally, the vertical transport properties of the patterned ZnO nanorod arrays were investigated through the microprobe station equipment, and the I-V measurement results indicated that the back-to-back Schottky contacts with different barrier heights were formed in dark conditions. Under UV light illumination, the patterned ZnO nanorod arrays showed a high UV light sensitivity, and the response ratio was about 10^4^. The controllable fabrication of patterned ZnO nanorod arrays and understanding their photoelectric transport properties were helpful to improve the performance of nanodevices based on them.

## Background

There is a growing interest in designing new architectures for enhancing the performance of photoelectric devices [1]. Due to the uniquely combined optical and electrical characteristics, ordered nanorod arrays have received considerable attentions for this goal [2]. Photoelectric devices based on ZnO, CdS, ZnS, InP, SnO_2_, and Si nanowires or nanorod arrays offer the advantages of enhanced light absorption, improved carrier collection efficiency, and longer lifetime for minority carriers compared to conventional planar photoelectric devices, which can find many applications from field emission devices, sensors, solar cells, nanogenerators to UV photodetectors with the significantly improved performances [3-9]. Among them, ZnO nanorod array is one of the most promising materials for photoelectric devices due to its large exciton binding energy (60 meV), versatile synthesis, high mechanical and thermal stabilities, and nontoxic n-type nature [10].

Recently, various synthetic methods have been developed for the growth and fabrication of vertically aligned ZnO nanorod arrays, which can be classified into two categories: vapor-phase and hydrothermal synthesis. The hydrothermal synthesis method is more favorable for the practical applications due to its low growth temperature, low cost, and good potential for scale-up. More importantly, this method avoids the usage of gold catalyst, which is commonly used in vapor-phase methods and may introduce the residual catalyst atoms into the ZnO rod arrays [11-13]. Based on the hydrothermal method, three dimensional (3D) ZnO hybrid architectures and “nanoforest” hierarchical ZnO arrays have been synthesized [14,15]. Theoretical and experimental works also have been carried out on the ZnO nanorod arrays based photoelectric devices and shown that the photoelectric response and charge carriers transport can be influenced greatly by the density and spacing of the ZnO nanorod arrays. Wang et al. report the field emission properties of ZnO arrays are correlative with the rod density [16]. Subsequently, Spencer et al. prove theoretically that varying the spacing will affect the sensing property of ZnO nanorods using the density functional theory [17]. Therefore, ZnO nanostructure arrays fabricated following a designed pattern, with a high degree of control in density and spacing is highly desirable. The development of nanofabrication techniques and equipments such as electron beam lithography (EBL), nanoimprint lithography, laser interference lithography, and nanosphere lithography provide us the potential opportunity to fulfill this goal [18].

However, precise control of these geometric parameters is rather challenging in practice. The e-beam lithography is the most reliable technique which can define the exact positions of the nanostructures with high precision. Therefore, the spacing distance between the patterned ZnO seed areas can be easily controlled in nanometer scales by using the EBL method. On the other hand, the hydrothermal growth technique of ZnO is versatile, large scale, and not confined to the inorganic substrate due to its low growth temperature as mentioned above. Therefore, combining these two methods together to fabricate patterned ZnO nanorod arrays with controllable geometric parameters is possible and attractive.

In this paper, patterned ZnO nanorod arrays with well-defined positions and spacing distance are fabricated onto Si substrate by a combination of EBL and hydrothermal growth process. The influences of spacing distance and growth time on the morphology of the nanorod arrays are also investigated. Finally, the transport properties of our ZnO nanorod arrays with suitable spacing distance and density are investigated.

## Methods

**Figure 1:**
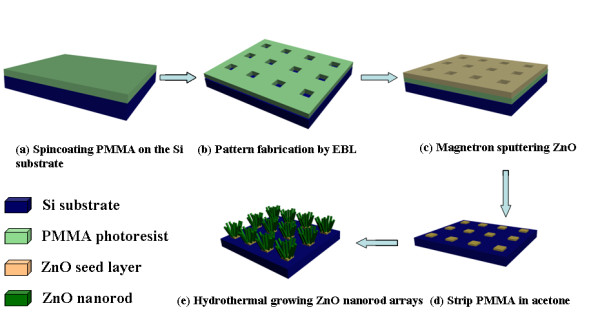
**Schematics of the experimental procedures of patterned ZnO nanorod arrays.** (**a**) Spincoating PMMA on Si substrate; (**b**) Pattern fabrication by EBL method; (**c**) Magnetron sputtering ZnO seed layer; (**d**) Strip PMMA in acetone solution; (**e**) Hydrothermal growth ZnO nanorods on the patterned areas.

Schematic diagram of the experimental procedures for the patterned ZnO nanorod arrays are shown in Figure 1. Si wafer is cleaned sequentially in ethanol and acetone for 20 min, respectively. A layer of polymethyl methacrylate (PMMA) is cast on the Si substrate at 2, 000 rpm (rounds per minute) for 40 s by spin coating methods as shown in Figure 1a. Then, the patterns are designed on PMMA by using the EBL methods as shown in Figure 1b. Reactive ion etching treatment (25 mTorr, 50 sccm, 50 W, 5 s) is used to remove the residual PMMA on the exposure Si substrate. ZnO seed layer (thickness about 40 nm) is deposited using magnetron sputtering method (Figure 1c), and after strip, the PMMA in acetone, patterned ZnO seed areas, are obtained on the Si substrate. (Figure 1d).

The hydrothermal method is used for the growth of ZnO nanorods on the patterned areas as shown in Figure 1e. For details, the Si substrate is immersed vertically in the nutrient solution of 0.035 M zinc nitrate [Zn(NO_3_)_2_ · 6H_2_O] and 0.65 M NH_3_. The reaction temperature is kept at 80°C with different reaction times. The morphologies of the samples are characterized by scanning electron microscopy (SEM) (JEOL, JSM-5600LV, Akishima, Tokyo, Japan), high-resolution transmission electron microscopy (high-resolution transmission electron microscopy (HRTEM) (JEOL JEM 2010). The current–voltage characteristics measurements are carried out on the probe station (Lake Shore) equipped with a Keithley 4,200 semiconductor characterization system (Cleveland, OH, USA). The light used in our experiment is an UV light source (*λ* = 350 nm) with an average power of 0.8 mW.

## Results and Discussion

**Figure 2:**
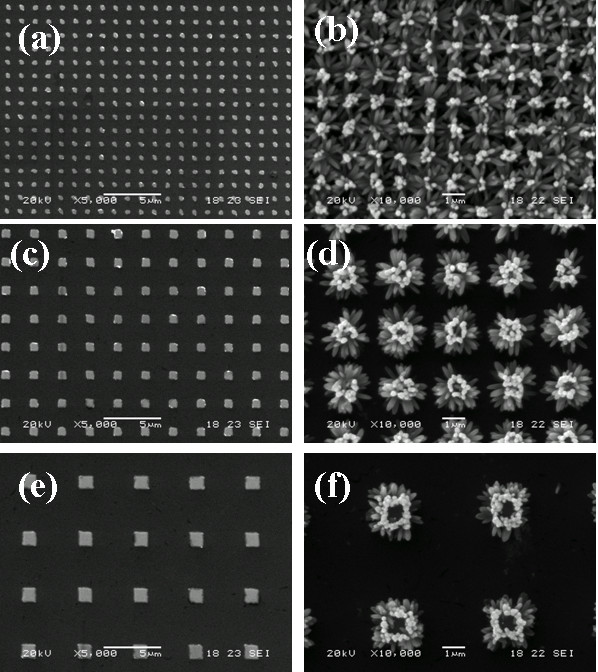
**The SEM image of ZnO dot area and nanorod arrays.** (**a**) The SEM image of ZnO dot area with 200 × 200 nm; (**c**) dot area is 500 × 500 nm; and (**e**) dot area is 1 × 1 μm. (**b**) The SEM image of ZnO nanorod arrays with 1-h hydrothermal reaction process on 200 × 200 nm seed areas; (**d**) on 500 × 500 nm seed areas; and (**f**) on 1 × 1 μm seed areas.

Figure 2 are the SEM images of ZnO nanorod arrays patterned by EBL method. Figure 2a,c,e is the ZnO dot patterns after peeling the PMMA layer from the Si substrate (a). The dot area is 200 × 200 nm, and the distance between each dot is 1 μm (c). The dot area is 500 × 500 nm, distance is 2 μm (e). The dot area is 1 × 1 μm, distance is 4 μm. Different dot areas with different spacing distances are obtained through EBL and subsequent magnetron sputtering techniques. Figure 2b,d,f is the nanorod arrays after hydrothermal process on the patterned ZnO seed areas at 80°C for 1-h reaction time. The results indicate that ZnO nanorod arrays are successfully obtained which confined to the ZnO seed areas. The length of the nanorod is about 1 μm with a diameter less than 100 nm. For the 200 × 200 nm ZnO seed areas with 1 μm spacing distance, the bundles of the nanorods are connected with each other without the edge confinement just like flowers on the Si substrate as shown in Figure 2b. While for the larger ZnO seed areas (500 × 500 nm) and longer spacing distance (2 μm), the nanorod patterns are separated with each other on the Si substrate, and the larger ZnO seed areas include more nanorods when compare with that of 200 × 200 nm areas. For the 1 × 1 μm ZnO seed areas, there are no nanorods that appear in the middle of the dot areas due to the short reaction time which is in accordance with the literature reports [19].

**Figure 3:**
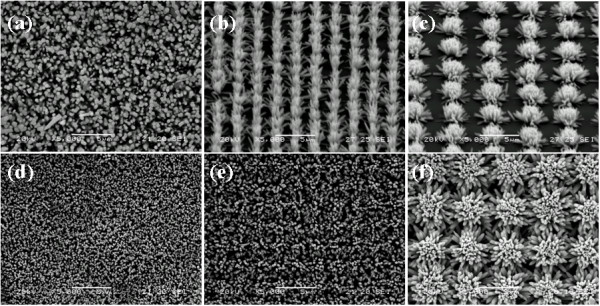
**The SEM image of ZnO dot area and nanorod arrays with growth time 1.5 and 2.0 h.** (**a**) The SEM image of ZnO nanorod arrays with growth time of 1.5 h on 200 × 200 nm seed areas; (**b**) seed areas are 500 × 500 nm; and (**c**) seed areas are1 × 1 μm; (**d**) The SEM image of ZnO nanorod arrays with growth time of 2.0 h on 200 × 200 nm seed areas; (**e**) 500 × 500 nm seed areas; and (**f**) 1 × 1 μm seed areas.

Since the growth time in the hydrothermal process is the main factor which can be used to control the morphology and density of ZnO nanorod patterns; therefore, the density variations of ZnO nanorod arrays are investigated for the different reaction times during the hydrothermal process. Figure 3a,b,c is the SEM images of ZnO nanorods with a 1.5-h reaction time on the same patterned areas used for the 1-h reaction time as shown in Figure 2. The patterns on 200 × 200 nm seed areas cannot be distinguished due to the longer reaction time, which make the nanorods bundles much longer to closeness with each other (Figure 3a). For the 500 × 500 nm seed areas as shown in Figure 3b, the nanorod arrays are vertical aligned on the Si substrate with 2 μm spacing distance. We should note that the ZnO nanorods at the edge of the pattern have a tendency to grow outward, whereas the nanorods at the central part of the patterns grow vertically to the substrate. Such effect appears more serious for the seed areas with a larger area size, which results in the hemispherical growth of ZnO nanorod array from the patterned areas just as shown in Figure 3c. With the growth time increased to 2 h, the patterns for 200 × 200 nm and 500 × 500 nm ZnO seed areas both disappeared as shown in Figure 3d,e. For the 1 × 1 μm ZnO seed areas with 4 μm spacing distance higher density of patterned ZnO nanorod arrays are obtained as display in Figure 3f. In addition, the diameters of the ZnO nanorods near the edge of the pattern are larger than that inside the pattern which can be seen in Figure 3f. The dependence of the diameters of the nanorods may be due to the effects of the density of neighboring nanorods which can hinder nanorods growth; and also, the nanorods at the edge have a greater precursor chemical source supply than are present for the high density ZnO nanorods in the central regions.

**Figure 4:**
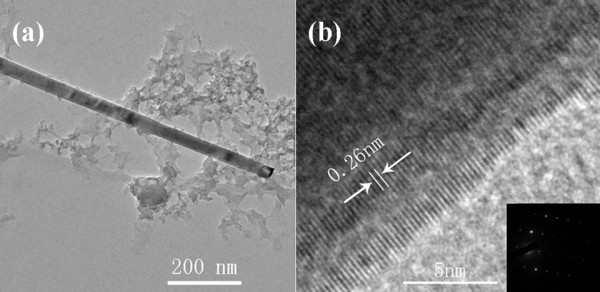
**The TEM and HRTEM images of ZnO nanorod.** (**a**) The TEM image of ZnO nanorod; (**b**) The HRTEM image of ZnO nanorod, and the inset image of Figure 4b is the diffraction pattern of ZnO nanorod.

Figure 4a,b is the transmission electron microscopy (TEM) and HRTEM images of individual ZnO nanorod, respectively. It shows the single nanorod with diameter less than 100 nm, and well-defined lattice fringe separation with 0.26 nm. It is also in agreement with the selected area electron diffraction results (the inset of Figure 4b) which indicates that the nanorod have a high quality single-crystalline structure and growth orientation along the [0001] direction.

**Figure 5:**
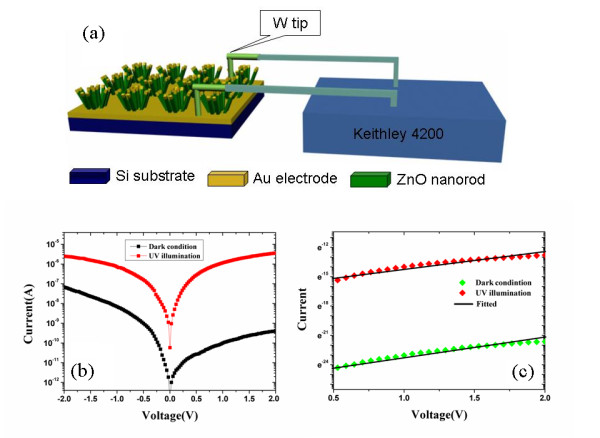
**Schematics of the apparatus used and the I-V characteristics of the ZnO nanorod arrays.** (**a**) The schematics of the apparatus used in our experiment; (**b**) I-V characteristics of the ZnO nanorod arrays measured in dark and under UV illumination; and (**c**) Fitted lnI versus V curve in dark and under UV illumination conditions.

The photoelectric transport properties of the patterned ZnO nanorod arrays are then investigated through the microprobe station equipment. Figure 5a is the schematics of the apparatus we used in our experiment. In this system, Au electrodes on the top of the nanorods and the Si substrate are designed through one-step deposition method on the 1.5-h growth time of the patterned ZnO nanorods in the 500 × 500 nm seed areas with 2-μm spacing distance. Figure 5b shows typical current voltage (I-V) characteristics of the fabricated ZnO nanorod structures measured in dark and under UV light illumination conditions. With applied bias (from −2.0 to +2.0) voltages, the curve exhibits asymmetrical nonlinear behavior with lower current value as several nA in dark conditions. Under UV light illumination, the current is increased greatly, and the curve exhibits from asymmetrical to symmetrical nonlinear I-V behavior. At 2 V applied bias voltage, the dark current is about 4.0 × 10^−10^ A, and the photocurrent is 3.0 × 10^−6^ A; the current contrast ratio of photocurrent to dark is about 10^4^. The nonlinear behaviors of ZnO nanorod have been reported in many literatures. Usually the nonlinear behavior is caused by the Schottky barriers formed between the semiconductor and the metal electrodes, and the shape of I-V curve depends on the heights of the Schottky barriers between the interface of metal and semiconductors [20-22]. The nonlinear I-V characters as the reverse current of Schottky diodes properties are usually described by the thermionic field emission model which has been developed by Padovani and Stratton [23]. In this model of Schottky diodes, the logarithmic plot of the current I as a function of the bias V give approximately a straight line of slope $\frac{q}{kT}-{\frac{1}{E}}_{0}$[24]. By fitting the I-V curves, we find that lnI is linear with V both for under dark and illumination conditions, just as shown in Figure 5c. Therefore, two back-to-back Schottky contacts are formed in Au-ZnO-Au structure. In this structure, the current is dominated by the reverse current of reverse biased Schottky barrier.

The asymmetrical I-V curves mean that the-two Schottky barriers heights are different in dark condition. In nanometer scales, the surfaces states on the semiconductor surface will play a crucial role in determining the electron transport properties of nanostructures. The previous studies by our group and others have demonstrated that the surface states on the ZnO nanostructure surface can dominate the photoelectric properties of the ZnO nanorod arrays, and different surface states which caused by O_2_ adsorption or vacancies on the surfaces of semiconductor nanowires will cause the asymmetrical behavior of I-V curves [25-30]. In this experiment, one end of the Au electrode is deposited on the top of the ZnO nanorods, and the other end is surrounding the patterned ZnO nanorods on the Si substrate. In the middle of the ZnO nanorod patterns, there might have no Au deposited because of the high density of the rod arrays. Therefore, different contact properties are formed between the two ends of the Au electrode, accordingly different surface states will formed between ZnO nanorod and Au electrodes. Different heights of the Schottky barriers at the interface of metal and semiconductor which dominate by the surface states will make the asymmetrical I-V curves in dark condition. Under UV light irradiation, the photogenerated electrons and holes are quickly separated through the strong local electric fields formed at the reverse bias Schottky barrier, resulting in the current increased greatly. Since the barrier height and build-in electric field of Schottky diodes are different, the separation efficiency of photogenerated electron–hole pairs in the depletion layer will be different [25]. The higher build-in electric field can separate more photogenerated holes to the surface of the ZnO nanorod, and the Schottky barriers height will be degraded larger also. As a result, the I-V curve changes from asymmetrical to symmetrical nonlinear I-V behavior under UV light illumination.

## Conclusions

In summary, we have demonstrated an effective approach for controllable fabrication of ZnO nanorod arrays with different geometric parameters through the combination of EBL and hydrothermal growth process. EBL is employed to fabricate the patterned ZnO seed layers with different areas and spacing distances with high precise, while a hydrothermal growth method is used to control the density and morphologies of ZnO nanorod arrays. This combined nanofabrication approach provide a possibility to integrate the patterned ZnO nanorod arrays into real devices. The vertical transport properties of the patterned ZnO nanorod arrays are investigated, and the I-V curve measurement indicates that the back-to-back Schottky contacts with different barrier heights are formed between the Au electrodes and ZnO nanorods in dark conditions. Under UV light illumination, the patterned ZnO nanorod arrays show a high UV light sensitivity, and the response ratio is about 10^4^. The controllable fabrication of patterned ZnO nanorod arrays and understanding their photo-electric transport properties are helpful to fabricate novel nanodevices based on them.

## Competing interests

The authors declare that they have no competing interests.

## Authors’ contributions

WSJ and SCC are the primary authors and carried out the experiments, characterization, and acquisition of data. CK participated in analysis and interpretation of data. DSX and ZZY participated in language modification. ZLD is the principal investigator who helped in analysis and interpretation of data, drafting of the manuscript, and revisions. All authors read and approved the final manuscript.
